# Dr. Trotter, on the Means of Destroying Contagion

**Published:** 1800-03

**Authors:** T. Trotter

**Affiliations:** Cawsand Bay


### *To the Editors of the Medical and Phyſical Journal.*

Gentlemen,

A Report having been in circulation for ſome time paſt in the Fleet, that a quantity of ſilk goods had been damaged and ſpoiled for ſale, by being fumigated with the nitrous acid vapour, under the idea of deſtroying peſtilential infection, which was ſuppoſed to exiſt in a ſhip in the river Thames, juſt arrived from a port in the Mediterranean,—ſhould this circumſtance have come within the knowledge of any of your Readers, who may be pleaſed to communicate the particulars to me, in any manner moſt agreeable, I ſhall conſider myſelf extremely obliged. Medical gentlemen, who may be acquainted with the ſhare which I have taken in this diſcuſſion, will readily believe that ſomething more than mere curioſity has prompted me to make the inquiry. The ſubject is at all times intereſting to profeſſional men; but particularly ſo during war, and while there is a dread of the plague being imported into the country, by our commerce. You muſt have heard, that it has been propoſed to fumigate the cloathing of ſeamen in this manner; ſuch an occurrence, therefore, at the chemical period of the eighteenth century, as exhibited on the London wharf, will be an uſeful beacon to naval ſervice.

In

In tracing the hiſtory of Fumigation to remote ages, I find it a matter of ſome difficulty to diſtinguiſh, whether it originally belonged to *Necromancy* or *Medicine*; but in thoſe times, a groſs ſuperſtition equally conducted the cure of diſeaſes and caſting out devils. In the Book of TOBIT, the inquiſitive reader will find a curious ſpecimen of this practice, where the *Devil* was expelled by the ſmoke and ſmell produced by burning the heart and liver of a fiſh, on the aſhes of perfumes. The context is highly entertaining. But *evil ſpirits*, in later times, have felt the omnipotence of fumigation. Lilly informs us, that "one Evans having raiſed a ſpirit, at the requeſt of Lord Bothwell and Sir K. Digby, and forgetting a *fumigation*, the ſpirit, vexed at the diſappointment, pulled him out of the circle, and carried him from his houſe in the Minories into a field near Batterſea Cauſeway."

It is worthy of remark, that the *oxygenated muriatic acid gas*, which, at the beginning of the war, was thought to do wonders againſt contagion, in the hands of French phyſicians, is already utterly forgot: yet the employment of this fume is ſaid to have given the firſt hint for the preparation of the nitrous vapour.* A late letter from Dr. Mitchill, of New York, acquaints me, that a Committee of Phyſicians, at Montpellier, had tranſmitted to the Secretary of the American States, a receipt for fumigation againſt the Yellow Fever. It recommends *ſmoking tobacco*, *burning pine-wood* in the ſtreets, and fumigating the chambers of the ſick with *caſcarilla!* The powder for fumigation, which was tried on the ten convicts at Moſcow, in 1770, who were expoſed to infected cloaths and bedding, was then ſaid to be effectual againſt the plague. It conſiſted of *leaves of juniper*, *juniper berries*, *ears of wheat*, *lign. guaiacum*, *ſalt petre*, *ſulphur*, *tar*, *and myrrh*. We ſuſpect this compoſition muſt have quickly fallen into diſrepute; for, as being of Ruſſian origin, it would have been employed to check the mortality which continues among the ſoldiers of that nation at Jerſey and Guernſey. It was a good idea of the Roman chief, who inſtituted the *Secular Games*, during the ravages of a plague at Rome; for thoſe amuſements might contribute to engage the attention of the multitude, and thus, in ſome meaſure, fortify the conſtitution againſt infection. But here alſo the ſpirit of fumigation prevailed, and *ſulphur* was ſerved out to the people for that purpoſe. Theſe *formulæ* are a few out of a number in my memorandum-book: you may obſerve their general character; like the *vinegar of the four thieves*, they are all

* Beddoes's Conſiderations on Airs, Part V.

all directed, by the pungent ſmell, to relieve the noſe. The common fate which has awaited the whole, exhibits a miſerable picture of medical faſhions, and holds up but poor conſolation to human nature, under the ſcourge of its moſt dreadful foe, impending peſtilence. I am,

GENTLEMEN,

Your moſt humble ſervant,

T. TROTTER.

